# Notices

**Published:** 1871-06

**Authors:** 


CHICAGO, April 1, 1871.

EDITORS CHICAGO MEDICAL JOURNAL:

I take pleasure in informing you that at the annual meeting of the Alumni Association of the Chicago Medical College, held March 14, 1871, the prize of $100 was awarded to DR. LYMAN WARE, class of 1866, for an essay entitled "Antagonisms between Opium and Belladonna." This essay, together with the President's Address, will be sent to all Alumni, who become members of the Association. You are respectfully urged to become a member, by sending your name and address, with one dollar, to the Secretary.

The Association has again voted $100 for a prize to that member of the Association who will present the best essay on some medical or surgical topic. Of the above sum, DR. N. S. DAVIS will donate $50. DR. LYMAN WARE donates $50 for a prize to that member of the Association who will present the second best essay on some medical or sugical topic. The competing essays must be placed in the hands of the Secretary of the Association, before the 15th February, 1872.

Each essay must be designated by a device or motto, and must be accompanied by a sealed envelope, bearing the same device or motto, containing the name and address of the author.

The essays will be examined by a committee, consisting of Drs. N. S. DAVIS, E. ANDREWS, H. A JOHNSON, and WALTER HAY; and the envelope accompanying the successful essay will be opened, and the name of the author announced, at the next annual meeting of the Association, in 1872.

Please forward your annual dues by return mail.

As I am unable to find the address of the following Alumni, you will confer a great favor by giving me information concerning them:

Charles Ashworth, class 1869; H. C. Barrett, class 1865; J. W. Barlow, class 1867; C. C. Bendeke, class 1869; R. W. Bower, class 1870; O. F. Bartlett, class 1862; J. Conant, class 1860; T. Cochrane, class 1865; G. M. Conville, class 1868; W. H. Crothers, class 1869; Wm. C. Chafee, class 1869; J. H. Curtiss, class 1869; E. Deane, class 1863; Wm. Deal, class 1869; S. H. Drake, class 1869; Lucius Dillie, class 1870; W. C. Griswold, class 1864; E. T. Greenleaf, class 1864; J. S. Gibson, class 1869; J. Hoke, class 1869; G. B. Hoblet, class 1869; J. H. Hudson, class 1870; C. D. H. Jones class 1860; C. T. Johnson, class 1866; J. H. Leitch, class 1862; J. E. Link, class 1865; J. R. Lane, class 1867; E. Y. Lawrence, class 1867; J. N. McLane, class 1863; S. McGiffin, class 1865; H. C. McCoy, class 1866; J. D. Morris, class 1863; H. G. Morgan, class 1868; A. S. Martin, class 1864; W. E. Morris, class 1866; J. Nicolai, class 1861; W. D. Plummer, class 1864; A. D. Rouse, class 1862; F. A. Reckard, class 1864; R. R. Resseguie, class 1867; David Robertson, class 1867; Meinard Risch, class 1869; Nelson Rinedollar, class 1869; S. W. Ransom, class 1870; W. D. Saxton, class 1865; Joseph Sterret, class 1869; W. M. Stratton, class 1870; W. H. York, class 1864; J. B. Woodson, class 1868.

S. A. McWilliams, M. D.,
Permanent Secretary and Treasurer, No. 166 State Street

---

### *Brainard Medical Society.*

The Society met in the Court House in Winamac, Ind., April 6, 1871, Dr. Cleland in the chair.

Drs. T. M. Goldsberry and A. S. Campbell of North Judson, and H. E. Pattison of Star City, were admitted to membership. Drs. Hoag, Cleland, Campbell and Thomas reported interesting cases in practice. The fifth annual election of officers was then had, which resulted as follows:

President—W. T. Cleland, Kewana.

Secretary—I. B. Washburn, Star City.

Treasurer—Wm. Kelsey, Monterey.

Censors—F. B. Thomas, J. B. Hoag, and A. S. Campbell.

Delegates to State Society—Drs. Washburn, Kelsey, Kittinger, Thomas, Glazebrook, and Hoag.

The Society adjourned to meet in Star City, Ind., July 6, 1871.

I. B. Washburn, *Sec.*

